# The Effects of Valence and Arousal on Associative Working Memory and Long-Term Memory

**DOI:** 10.1371/journal.pone.0052616

**Published:** 2012-12-26

**Authors:** Heiko C. Bergmann, Mark Rijpkema, Guillén Fernández, Roy P. C. Kessels

**Affiliations:** 1 Radboud University Nijmegen, Donders Institute for Brain, Cognition and Behaviour, Nijmegen, The Netherlands; 2 Radboud University Nijmegen Medical Centre, Department for Cognitive Neuroscience, Nijmegen, The Netherlands; 3 Radboud University Nijmegen Medical Centre, Department of Medical Psychology, Nijmegen, The Netherlands; University of Bologna, Italy

## Abstract

**Background:**

Emotion can either facilitate or impair memory, depending on what, when and how memory is tested and whether the paradigm at hand is administered as a working memory (WM) or a long-term memory (LTM) task. Whereas emotionally arousing single stimuli are more likely to be remembered, memory for the relationship between two or more component parts (i.e., relational memory) appears to be worse in the presence of emotional stimuli, at least in some relational memory tasks. The current study investigated the effects of both valence (neutral vs. positive vs. negative) and arousal (low vs. high) in an inter-item WM binding and LTM task.

**Methodology/Principal Findings:**

A five-pair delayed-match-to-sample (WM) task was administered. In each trial, study pairs consisted of one neutral picture and a second picture of which the emotional qualities (valence and arousal levels) were manipulated. These pairs had to be remembered across a delay interval of 10 seconds. This was followed by a probe phase in which five pairs were tested. After completion of this task, an unexpected single item LTM task as well as an LTM task for the pairs was assessed. As expected, emotional arousal impaired WM processing. This was reflected in lower accuracy for pairs consisting of high-arousal pictures compared to pairs with low-arousal pictures. A similar effect was found for the associative LTM task. However, the arousal effect was modulated by affective valence for the WM but not the LTM task; pairs with low-arousal negative pictures were not processed as well in the WM task. No significant differences were found for the single-item LTM task.

**Conclusions/Significance:**

The present study provides additional evidence that processes during initial perception/encoding and post-encoding processes, the time interval between study and test and the interaction between valence and arousal might modulate the effects of “emotion” on associative memory.

## Introduction

The likelihood of remembering a stimulus or an event is modulated not only by how the information is encoded (intentional vs. incidental) and how memory is tested (e.g., free recall vs. recognition memory) but also critically by their emotional content. Whereas this effect was originally referred to as the “emotional enhancement effect on memory” (e.g. [1]; see review by [2]), it has become clear that emotion can facilitate, yet also impair memory. This is dependent on how emotion is manipulated and upon how, when, and what kind of memory is tested. More specifically, memory performance may be a function of whether memory for single items or the relation between two or more component parts (i.e., relational memory) is tested [3], [4], which aspects in a relational memory paradigm are tested (e.g., the emotional or non-emotional part of a scene) [5]–[9], and of the length of the delay between study and test, particularly whether the task is administered as a working memory (WM) or long-term memory (LTM) task [10]. In addition, both valence and arousal levels of the stimuli or events [11]–[13] and how “emotion” is manipulated (e.g., through mood induction, by manipulation of the emotional content of the to-be-remembered stimuli or of distracting stimuli) are all critical determinants of the accuracy with which an event is remembered.

It has been noted that much of the evidence in favor of the “original” emotional enhancement effect of memory has been derived from studies examining memory for individual items only. However, single-item memory tasks lack the typical relational and associative nature of “real-life” episodic memories [4], [14], [15]. Indeed, emotional memory enhancement does not always extend to relational memory tasks, at least not in a straightforward way. That is, the effect of emotion hinges critically on what is tested. For example, when more complex *scenes,* which consist of an arousing item and a neutral background, are encoded and tested in a subsequent episodic memory task, memory for emotionally arousing central items seems to be better than for emotionally neutral items. In contrast, memory performance for the details of the background shows the opposite pattern; with impaired memory when the background is presented together with an emotionally arousing central item in comparison to when it is presented with a neutral central item [5], [6], [8], [9], [16], [17]. This effect is also nicely reflected in the well-known weapon-focus effect, in which people are more apt at remembering the weapon in a crime (real or simulated) in much detail, but are more likely to forget other contextual details [18]. As an explanation, it has been suggested that the arousal level of emotional stimuli modulates and biases the perceptual competition, with highly arousing stimuli being more likely to capture the attention [19]–[23] and thus benefiting from prioritized processing [24], [25]. Consequently, they will be more likely to be remembered in a subsequent memory task, possibly also depending on whether the attention–grabbing stimulus is task-relevant [10], [26], [27]. At the same time, this competition bias on the perceptual level may result in less-arousing stimuli being less attended to and, thus, more likely to be forgotten (i.e., not consolidated into LTM).

Hence, the type of the memory test (single item vs. relational memory test) and the aspects of an event that are tested (e.g., central or peripheral items or the context) are critical for determining the accuracy with which an event is remembered. With this said, however, different types of relational memory have been distinguished [28], [29] and it appears that the effect of emotion on memory may depend upon what kind of relational memory task is administered (see [14]): Recent studies on emotional arousal and relational memory have mainly employed intra-item relational memory binding tasks, for example, object-color binding tasks [30]–[33] or object-location paradigms [4], [15], [30], [34], [35]. The majority of these studies showed increased performance on an unexpected subsequent recognition memory task for stimulus-color as well as stimulus-location associations when the stimulus was emotionally arousing compared to non-arousing, neutral stimuli. Interestingly, the very few studies using paired-associate memory tasks, where the relationship between two or more objects has to be remembered (i.e., inter-item binding paradigms), showed the opposite pattern. For example, paired-associate memory tasks in which the associated word had to be generated to a cue word demonstrated lower performance for associates of emotional rather than neutral cue words, hence indicating *impaired* performance on inter-item binding paradigms for emotional stimuli [36]–[38] (see [39] for contradicting evidence; it has been argued however, that this may be due to enhanced item memory rather than associative memory *per se* [3]). Another study addressed the issue of possible differential effects of emotion on intra- and inter-item binding tasks empirically and found differential effects of valence for these two tasks, depending on whether participants were instructed to visualize neutral-neutral or neutral-emotional word pairs as an integrated unit or to visualize them separately from one another [40]. To our knowledge, however, no inter-item binding studies exist that have used non-verbal stimuli, which are thought to be more ecologically valid and to be processed more efficiently [41].

Finally, the interval between study and test is important in determining the effect of emotion. Not only is there some evidence across studies that emotional enhancement increases as retention intervals increase [11], [42]–[44], but differential results might also be expected for WM as compared to LTM tasks. Most emotional memory studies in which the emotional content of the stimuli rather than the mood of the participants was manipulated were administered as LTM tasks and there is a clear lack of studies using WM paradigms. There is some evidence however that emotional content might differentially affect performance on WM and LTM. For example, Kensinger & Corkin (2003; [32]) conducted five experiments, in which they assessed different WM paradigms (memory updating, word span, n-back task), as well as subsequent LTM tasks that were typically administered one day after the WM task. Although their tasks did not rely on relational memory, the results indicated that performance on the WM tasks was not affected by the emotional content of the stimuli. In contrast, performance on the different LTM tasks showed the well-established emotional memory effect with higher accuracy for emotionally arousing in comparison to non-arousing stimuli. Another study administered a delayed-match-to-sample (DMS) WM task in which each trial consisted of four serially presented items that had to be remembered, along with their respective locations on the screen across a 7s-delay interval (i.e., an intra-item binding WM task [15]). Stimuli were drawn from the International Affective Picture System (IAPS; [45]). These stimuli were selected based upon their arousal levels and divided into three categories: non-arousing (neutral), low arousal and high arousal. The authors reported an “inverted” emotional effect: the higher the arousal level of the stimulus, the *less* likely it was to be correctly processed together with its corresponding locations in the WM task. Interestingly, these results seemed to contradict studies in which memory was not tested immediately but after some delay; here performance seemed to rely on LTM instead of WM. In most of the “LTM studies” the opposite pattern has been reported, with better performance on the intra-item binding of emotionally arousing stimuli than for the binding of neutral/non-arousing stimuli on different kinds of relational memory tasks.

Next to these arousal effects, it has also been suggested that the valence of an event (i.e., whether it is pleasurable or aversive) may modulate memory effects [11]. For example, it has been reported that negative items are more likely to be remembered in detail than emotionally neutral or positive items, at least in young adults [17], [46]. However, only very few studies exist that investigated the effects of both valence and arousal.

To the best of our knowledge, no inter-item memory binding tasks have been reported that used non-verbal stimuli and combined a WM and a LTM test in one single experimental design. In addition, most studies did not separate arousal effects from the effect of valence. With the previous statement in mind, the present study combined an inter-item WM-binding with an unexpected subsequent LTM task, using identical stimuli and similar task requirements for these two tasks. This permits the investigation of the effect of valence and arousal on both WM and LTM, using pictorial stimuli. A DMS task was employed in which emotionally neutral stimuli were always paired with a second stimulus of which the emotional content was manipulated. Based on previous studies and the object-binding theory [14] we predicted that high-arousal stimuli would increase attention for the stimulus content, thereby producing a cost for the required binding process. Consequently, picture pairs containing high-arousal pictures were hypothesized to be less likely to be correctly processed in WM than picture pairs consisting of less arousing stimuli. In addition, as there is some evidence that not only encoding-related but also post-encoding or consolidation processes affect the outcome in episodic LTM tasks and based upon previously reports on impaired emotional memory on paired-associate tasks, we hypothesized similar effects for the unexpected associative LTM task (i.e., a detrimental effect of arousal). Finally, the attention bias towards high-arousal stimuli may be expected to be reflected in better single-item memory as opposed to memory for less arousing stimuli. This hypothesis was tested in a single-item LTM task.

## Methods

### Participants

Fourty-three female students (mean age 21.34 years, SD 2.31, range 18–26 years) of the Radboud University Nijmegen participated in the experiment. All participants had a score of 10 or lower on the Beck Depression Inventory (BDI; Beck, Ward, Mendelson, Mock, & Erbaugh, 1961). Participants had normal or corrected-to-normal vision and were compensated for their participation with either course credit points or 10 Euros. Participants were told beforehand that the experiment contained potentially highly arousing emotional pictures and gave written informed consent according to the local ethics committee of the faculty of social sciences of the Radboud University Nijmegen, and the declaration of Helsinki. We only included female participants in the current experiment for two reasons: Firstly, it has been shown that females tend to be more responsive to emotional stimuli and might process them more automatically than males [47]. Secondly, women tend to rate a greater proportion of pictures as highly arousing [48] and, consequently, the individually perceived arousal between different stimulus types may differ to a larger extent for females than for males.

### Material

#### Stimuli

The stimuli for the WM and LTM task were drawn from the IAPS [45]. The IAPS is a stimulus set of colour pictures, which communicate their affective quality relatively quickly. We selected IAPS stimuli on the basis of their valence (positive vs. negative vs. neutral) and arousal (low vs. high) ratings. Since neutral stimuli are, by definition, not arousing, the pictures were compiled into five different categories: (1) high-arousal positive, (2) low-arousal positive, (3) high-arousal negative, (4) low-arousal negative and (5) neutral pictures. In total 250 IAPS pictures were selected, 125 low arousal/neutral pictures (Valence: *M* = 5.14, *SD* = 2.06, Arousal: *M* = 3.71, *SD* = 2.06), as well as additional 25 pictures per category. Efforts were made to match the stimuli across categories with respect to the content (e.g., presence of people, animals). In addition, since the IAPS stimulus set contains many photos of the same object type (e.g., many snakes and spiders), we did not select more than three of each type and care was taken that all stimuli could be discriminated well from each other. To check whether arousal and valence were accordingly matched, a one-way MANOVA with the six levels of Category as between-subjects factor was run on the Arousal and Valence ratings of female participants as provided by Lang, Bradley and Cuthbert [45]. The ratings differed in the intended and manipulated way, for example: positive high-arousal pictures did differ from negative high-arousal pictures in terms of their valence (*p*<.0005) level but not in terms of arousal levels (*p* = .95). In addition, arousal levels of the neutral pictures was lower than those for both positive (*p* = .02) and negative low-arousal (*p* = .004) stimuli, whereas the latter two did not differ significantly from each other (*p* = .69; see Table 1).

**Table 1:** Mean (SD) of valence (V) and arousal (A) ratings for the five different stimuli categories as provided by Lang et al. [45].

		Valence		
		Positive	Negative	Neutral
Arousal	Low	V = 7.72 (0.49)	V = 3.05 (0.61)	V = 5.19 (0.64)
		A = 4.05 (0.33)	A = 4.14 (0.30)	A = 3.65 (0.93)
	High	V = 7.49 (0.42)	V = 2.90 (0.46)	
		A = 6.30 (0.41)	A = 6.28 (0.40)	

### Procedure

#### DMS/WM task

The delayed-match-to-sample task was a five-pair associative WM task. The encoding phase of each trial consisted of five consecutively presented picture pairs, with each pair being shown for 2.0 s and separated from each other by a 0.5 s ISI, during which a white fixation cross on a black background was presented. Each pair consisted of one emotionally neutral picture and one picture of which the emotional content was manipulated (hereafter referred to as the “emotional” picture, which could be a high-arousal positive, low-arousal positive, high-arousal negative, low-arousal negative or another neutral picture). The location of the two pictures (i.e., on the left or right side of the screen) was randomized. In addition, the emotional content of the emotional picture varied across, but not within, trials. Participants were asked to remember the five picture pairs over a delay phase of ten seconds, whilst looking at a fixation cross. The following probe phase consisted of five consecutively presented picture pairs, each shown for 2 s. For each pair, participants were asked to indicate whether it matched one of the five pairs of the encoding phase of that trial. Participants were instructed to respond as fast as possible without sacrificing accuracy. Non-matches were intra-trial re-arranged pairings so that judgements could not be based upon the familiarity of the individual items. Moreover, the location of the two pictures was again randomized for each pair so that the same picture could be either in the same location as during the encoding phase or not. In total, 20 trials were administered, yielding 100 decisions to be made or 20 per condition/category. Per condition, 10 of the 20 test pairs were matches and the remaining 10 were non-matches. On any given trial, there were only two or three matches. Participants were unaware of this restriction however. Preceding the WM task, participants received written instructions and were administered two practice trials.

#### Single-item LTM task

After completion of the WM task, participants received instructions for the unexpected single-item LTM task. The task was a yes/no recognition memory task and each trial consisted of an emotional picture that either was or was not presented during the WM task (“old” or “new”, respectively). Only emotional pictures that were presented as a match in the WM probe phase were used for this task and analyses were restricted to items that were (as a pair together with the non-emotional stimulus) correctly processed during the WM task. This was done in order to correct for WM performance and to ensure as reliably as possible that results on the LTM task were not contaminated by performance on the WM task. Participants were instructed to rate each stimulus on a confidence scale that ranged from 1 (“definitely not seen during the WM task”) to 6 (“definitely seen”) by pressing corresponding buttons on the keyboard.

#### LTM binding task

**Figure 1:**
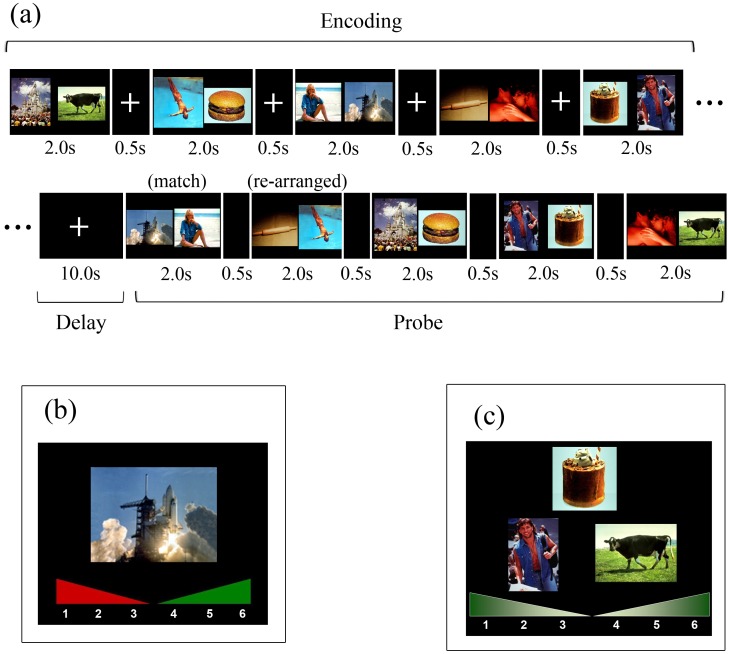
Schematic overview of the delayed-match-to-sample-task (a) and the two long-term memory tasks (b and c). Panel (a) shows a schematic representation of one trial of the delayed-match-to-sample task (with high-arousal stimuli). (b) One example of a trial of the single item LTM task. In the actual experiment, the confidence scale consisted of the scale, the numbers and a short explanation underneath each number (e.g. “definitely not seen” beneath “1″; “definitely seen” beneath “6” etc.). (c) One trial of the subsequent LTM Binding task. In the actual experiment, the confidence scale consisted of the scale, the numbers and a short explanation underneath each number (e.g. “definitely seen with the left picture” beneath “1″; “definitely seen with the right picture” beneath “6” etc.).

The effect of valence and arousal on associative LTM was assessed with an unexpected subsequent recognition memory task, administered after the single item LTM task (see Fig. 1). Each trial consisted of one emotional picture that was depicted in the upper part of the screen and two non-emotional pictures that were presented below and next to each other. The location of these two stimuli (i.e., left or right) was randomized. One of these non-emotional pictures was paired with the emotional picture during the encoding phase and also probed during the WM task (i.e., the pair in question was presented twice during the WM task). The second non-emotional picture was also presented during the WM task but was part of a re-arranged pair during the WM probe phase. Hence, all stimuli were presented twice during the WM task and judgements could not be based upon familiarity of one of the individual items. The participants’ task was to choose the correct pairing and could rate each decision on a confidence scale that ranged from 1 (“definitely seen together with the left picture”) to 3 (“not sure, but maybe seen with the left picture”) and from 4 (“not sure, but maybe seen with the right picture”) to 6 (“definitely seen together with the right picture”). The confidence scale was depicted at the lower part of the screen throughout the LTM task. The task was self-paced and test pairs were separated from each other by a 500 ms ISI. See Fig. 1 for more details.

#### Statistical analyses

The data were submitted to separate Repeated Measures ANOVA’s with either Corrected Recognition (Hit Rate – False Alarm Rate) or A′ as dependent variables. Because of the very low number of false alarms (e.g., on average, the false alarm rate for the single item LTM task was 2%), A′ instead of d’ was chosen as a signal-detection measure. A′ prime was calculated as follows: A′ = 0.5+ (HR − FAR)(1+ HR − FAR)/4HR(1 − FAR), where HR is the individual Hit Rate and FAR the individual False Alarm Rate. Since the binding LTM task was a two-alternative forced choice task, no false alarm could be defined, therefore “Proportion Correct” was used as the outcome measure.

## Results

### DMS/WM Task

**Figure 2:**
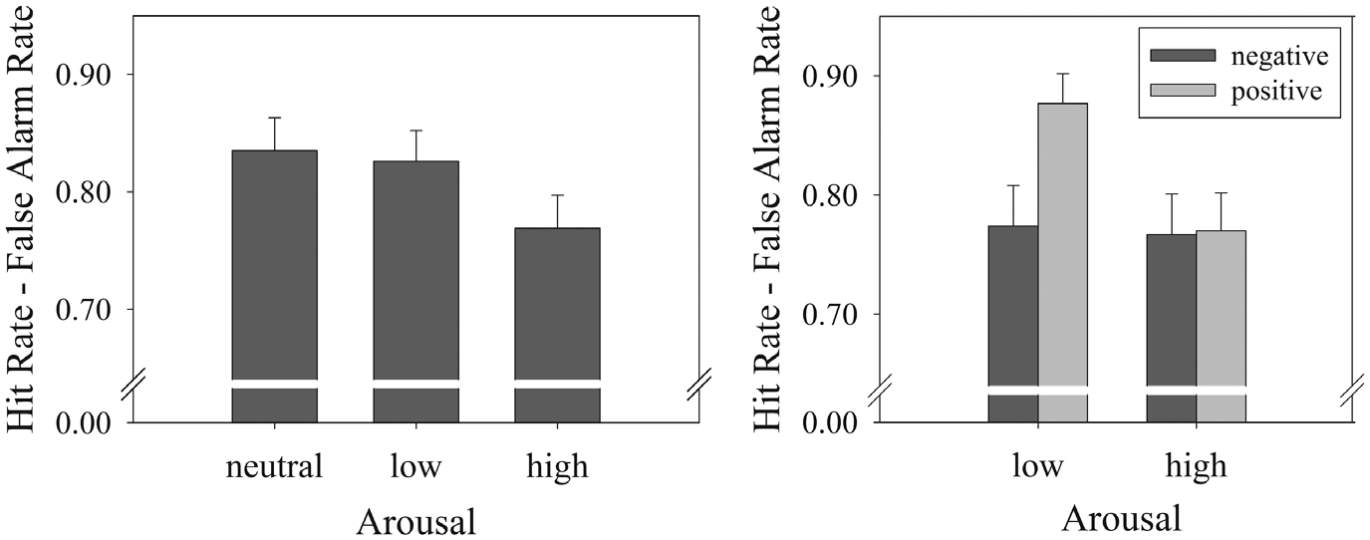
Bar graphs of the results of the delayed-match-to sample (WM) task. (a) Hit – False Alarm Rates for pairs consisting of one neutral and either another neutral or a low- or high arousal picture. (b) Corrected Recognition when considering Valence levels. Particularly pairs containing a low-arousal positive picture were more likely to be correctly remembered. Error bars represent the standard error of mean.

First, a Repeated Measures ANOVA with Arousal (neutral vs. low vs. high) was conducted on the ‘Hit Rate – False Alarm Rate’ of the WM task (table 2 provides a summary of hit and false alarm rates as well as sensitivity measures for all three administered memory tasks). This analysis yielded a main effect of Arousal, *F*(2,41) = 4.27, *p* = .02, η*_p_* ^2^ = .17). Post-hoc *t*-tests showed that pairs consisting of high-arousal pictures (*M* = 0.77) were less likely to be correctly processed than pairs containing neutral (*M* = .84, *p* = .006) or low-arousal pictures (*M* = .83, *p* = 0.05). See Fig. 2 (left panel) for more details.

**Table 2:** Hit (HR), false alarm rates (FAR), the sensitivity measure A’, and Proportion Correct (PR) as a function of task and condition.

	Condition				
	neutral	positive/low	positive/high	negative/low	negative/high
WM	HR = .91	HR = .93	HR = .88	HR = .89	HR = .89
	FAR = .07	FAR = .05	FAR = .10	FAR = .11	FAR = .12
	A’ = .95	A’ = .97	A’ = .93	A’ = .93	A’ = .93
LTM single	HR = .89	HR = .88	HR = .88	HR = .90	HR = .86
	FAR = .06	FAR = .01	FAR = .01	FAR = .04	FAR = .01
	A’ = .96	A’ = .97	A’ = .97	A’ = .96	A’ = .96
LTM binding	PR = .89	PR = .91	PR = .83	PR = .88	PR = .85

A similar analysis was run with A′ as the dependent measure. This time, the main effect of Arousal just failed to reach significance (*F*(2,41) = 2.80, *p* = .07, η_p_^2^ = .12, MSE = 0.003). Only post-hoc *t*-tests confirmed that pairs consisting of low-arousal pictures were better processed (*A*′ = 0.95) than pairs containing high-arousal pictures (*A*′ = 0.93, *p* = .02), while there was only a trend towards neutral-neutral picture pairs being better processed (*A*′ = .95) than pairs with high-arousal pictures (*p* = .09).

Omitting the neutral/neutral pairs, another Repeated Measures ANOVA with Valence (positive vs. negative) and Arousal (low vs. high) as within-subjects factors and ‘Corrected Recognition‘ (Hit Rate – False Alarm rate) as dependent variable was run (Fig. 2, right panel). Both a significant main effect of Valence (*F*(1,42) = 5.15, *p* = .028, η*_p_* ^2^ = .109, *MSE* = 0.023) and of Arousal (*F*(1,42) = 8.52, *p* = .006, η*_p_* ^2^ = .169, *MSE* = 0.016) were obtained. These main effects were qualified by a significant interaction between these two factors, (*F*(1,42) = 4.75, *p* = .035, η*_p_* ^2^ = .102, *MSE* = 0.023). Post-hoc analyses showed that this was driven by a better memory for pairs containing low-arousal positive pictures (*M* = .877) relative to the other three possible combinations (all *p*-values ≤.002) whereas the latter three did not differ significantly from each other (all *t*’s <1).

A similar analysis with A′ as the dependent variable revealed the same main effects of Arousal (*F*(1,42) = 5.20, *p* = .03, η*_p_* ^2^ = .11, *MSE* = 0.003) and Valence (*F*(2,41) = 4.82, *p* = .03, η*_p_* ^2^ = .10, *MSE* = 0.018). The interaction between these two factors, however, just failed to reach significance, (*F*(2,41) = 3.72, *p* = .06, η*_p_* ^2^ = .08, *MSE* = 0.004). As can be seen in Table 2, the pattern looks very similar to the analysis with the corrected recognition as dependent measure; with pairs consisting of low-arousal positive pictures being most likely to be correctly processed. See Fig. 2 for more details.

### LTM Task – Single Item

**Figure 3:**
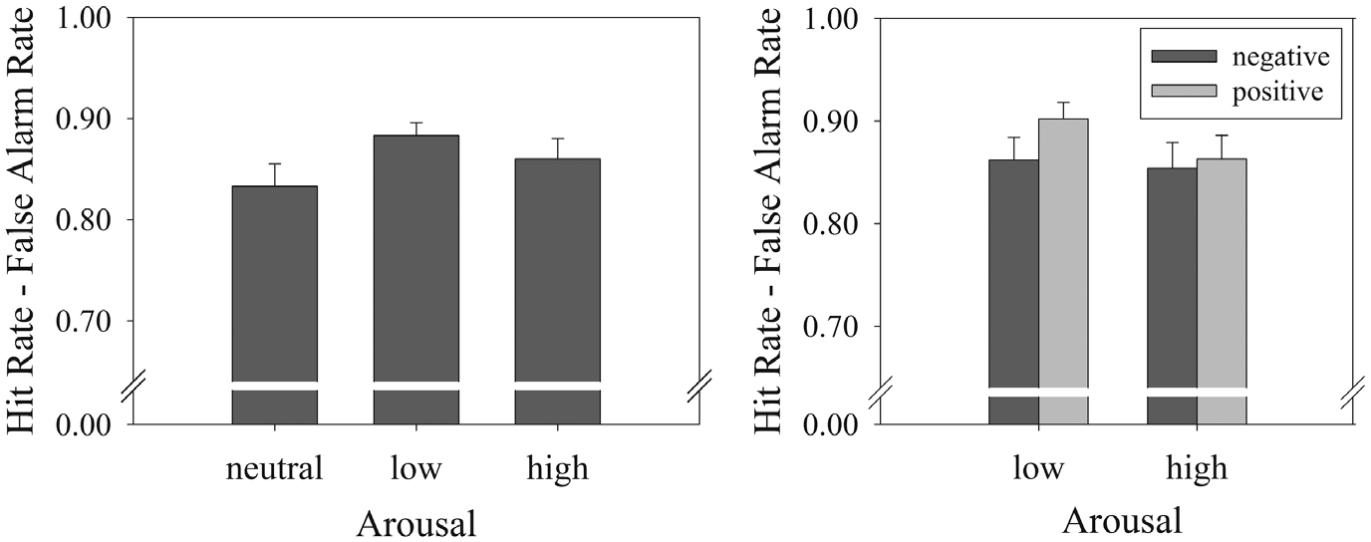
Bar graphs of the results of the single-item LTM task. (a) ‘Hit – False Alarm Rates’ for neutral, low-arousal and high-arousal items in the single item LTM task. No significant differences were found. (b) Corrected Recognition when considering Valence levels. No statistically reliable differences were found.

To ensure that performance on the WM task did not confound the results of the LTM tasks, only ‘hits’ of the WM task were considered in these analyses. Thus, incorrect trials of the WM task were excluded from analyses when computing the hit rate of the LTM tasks. A LTM “hit” was defined as correctly endorsing a picture as “old” with high confidence (i.e., 5 & 6 ratings). False alarms were defined as incorrectly identifying a picture as “old” with high confidence (i.e., 5 & 6 ratings). An initial Repeated Measures ANOVA with Arousal (neutral vs. low vs. high) as within-subjects factor and ‘Corrected Recognition’ as dependent variable did not reveal a main effect of Arousal (*F*(2,41) = 2.26, *p* = .12, η*_p_* ^2^ = .099) (see Fig. 3, left panel). The analogous analysis with A’ as the dependent measure did not yield a main effect of Arousal either (*F*(2,41) = 2.12, *p* = .13, η*_p_* ^2^ = .09, *MSE* = 0.003).

Subsequently, a 2 (Valence: positive vs. negative) × 2 (Arousal: low vs. high) Repeated Measures ANOVA was conducted to evaluate the effect of both Valence and Arousal on single item LTM (Fig. 3, right panel). This analysis did neither reveal main effects of Valence (*F*(1,42) = 1.68, *p* = .20, η*_p_* ^2^ = .039), Arousal (*F*(1,42) = 1.61, *p* = .21, η*_p_* ^2^ = .037) nor an interaction effect (*F*(1,42) = 1.13, *p* = .29, η*_p_* ^2^ = .026). The analysis with A’ revealed identical results, with no main effects of Arousal (*F*<1) and Valence (*F*(1,42) = 2.13, *p* = .15, η*_p_* ^2^ = .05, *MSE* = 0.001), and no significant interaction (*F*(1,42) = 1.46, *p* = .23, η*_p_* ^2^ = .03, *MSE* = 0.001). See Fig. 3 for more details.

### LTM Task – Binding Condition

**Figure 4:**
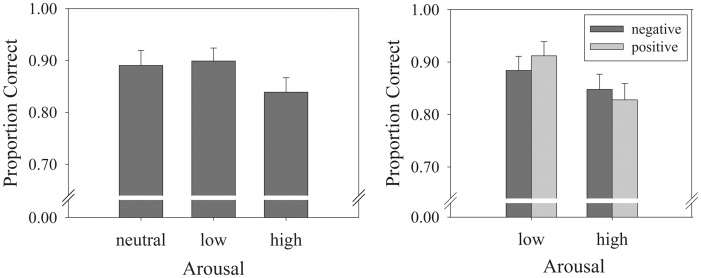
Bar graphs of the results of the associative LTM task. (a) Proportion Correct for pairs consisting of one neutral picture and another neutral or a low- or high arousal picture. Pairs consisting of high-arousal picture were less likely to be remembered in the LTM task. (b) Proportion Correct when considering Valence levels. Particularly pairs containing high-arousal positive picture were less likely to be remembered. Error bars represent the standard error of mean.

Again a “hit” was defined as correctly endorsing pairs as intact with high confidence (1 & 2 ratings or 5 & 6 ratings, depending on the location of the matching picture). An initial Repeated Measures ANOVA with Arousal (neutral vs. low vs. high) as within subjects factor and ‘Proportion ‘Correct’ as dependent variable was conducted. Again, a main effect of Arousal was obtained, *F*(2,41) = 6.30, *p* = .004, η*_p_* ^2^ = .235, *MSE* = 0.048 (Fig. 4, left panel). As in the WM task, stimulus pairs containing low-arousal pictures (*M* = .89) were equally well remembered than neutral/neutral picture pairs (*M* = .88, *p* = . 42), but both stimulus types were better recalled than pairs consisting of high-arousal pictures (*M* = .84; *p* = .001 and *p* = .01, respectively).

Omitting the neutral/neutral pairs, a 2 (Valence: positive vs. negative) × 2 (Arousal: low vs. high) Repeated measures ANOVA was conducted (Fig. 4, right panel). Again, a main effect of Arousal was found, *F*(1,42) = 12.94, *p* = .001, η*_p_* ^2^ = .236, *MSE* = 0.011. Pairs consisting of low-arousal pictures (*M* = 89.8%) were significantly better remembered than pairs consisting of high-arousal pictures (*M* = 84.0%). Moreover, neither a main effect of Valence (*F*<1), nor an interaction effect (*F*(1,42) = 1.79, *p* = .19, η*_p_* ^2^ = .041, *MSE* = 0.012) was obtained.

## Discussion

To our knowledge, the present paper is the first to investigate the effects of valence and arousal for inter-item WM binding, single-item LTM and inter-item binding LTM tasks, using the same stimuli and highly similar task requirements for WM and LTM tasks. In the administered WM (DMS) task, picture pairs consisting of one neutral picture as well as one picture of which the emotional quality was manipulated (the emotional picture), were to be remembered in each trial and tested after a short delay of 10 seconds. After completion of the WM task, memory for the emotional picture, as well as the pairing was tested again in a single item LTM and a binding LTM task. Results showed a “reversed” arousal effect for the WM binding task; pairs consisting of low-arousal or two neutral stimuli were *more* likely to be correctly processed than pairs consisting of high-arousal pictures. Taking affective valence into account, it was shown, however, that the advantageous effect of low-arousal stimuli was specific for positive valence. Similar “detrimental” arousal effects were found for the LTM binding task. On this occasion, however, no interaction effect was found and pairs consisting low-arousal pictures were better remembered than pairs with high-arousal stimuli, irrespective of their affective valence. Finally, no significant effects were found for the single item LTM task.

### Working Memory

The “detrimental” effects of arousal on the administered inter-item WM binding task are in line with our hypotheses and with previous studies that, when compared across studies, indicated differential effects for inter- vs. intra-item WM binding tasks. Our data also support the object-based framework proposed by Mather (2007) that explains the differential results on these two types of tasks with (additional) attentional processes required for inter-item WM binding tasks compared to intra-item binding tasks: In this view, arousing stimuli are thought to automatically capture the attention and that this increased attention towards arousing stimuli is disengaged more slowly when compared to neutral stimuli [49], [50]. As a consequence, the attended item and its subcomponents are thought to be perceived and bound into one coherent object representation, whereas other, less-attented, objects and, thus, object-scene or object-object interrelationships tend to be ignored [51]. Hence, the focus on arousing stimuli may leave insufficient attentional resources that would be required for binding inter-item relationships [52] and thus might be detrimental for inter-item associations but be beneficial for memory for intra-item associations (see also [53]).

Interestingly, WM was compromised not only for pairs containing high arousal pictures; a significant valence by arousal interaction showed that pairs with negative low-arousal pictures were less likely to be correctly processed. It may be hypothesized that negatively arousing stimuli in general tend to attract attention, thereby producing a cost for the binding process. Enhanced vigilance for negative and, hence, potentially threatening objects in general would not only make sense from an evolutionary point of view (the precise level of threat may be secondary), but also finds empirical support in the literature. For example, it has been shown that negative words slow down lexical decisions relative to positive words [20], [54], [55]. Slower responses to negative stimuli relative to positive or neutral stimuli have also been demonstrated in other paradigms, typically interpreted as being due to the automatic vigilance towards negative stimuli [56], [57] (however, see [58]).

### Long-term Memory: Single-item Task

The current study did not confirm our hypothesis that high-arousal pictures are more likely to be correctly remembered when tested individually. Possibly, the time interval between the WM and LTM task may have been too short to reveal significant differences. There is some evidence that the “typical” emotional enhancement effect increases with longer retention intervals [11], [42]–[44] and it is therefore possible that the hypothesized arousal effect might have been obtained if the interval between the WM task and the single item LTM task had been increased. In addition, performance levels on the single-item memory task were high (although only two participants scored at ceiling), possibly occluding the hypothesized effects. One solution to these two problems might be to increase the number of trials or to increase the similarity between target and distracting stimuli in the LTM task (as, for example, has been done, in [4]).

### Long-term Memory: Binding

The LTM binding task revealed mostly similar arousal effects as the WM task. Again, picture pairs consisting of low-arousal pictures were more likely to be remembered than pairs consisting of high-arousal pictures. However, whereas the arousal effect on the WM task may be explained by the previously discussed arousal-biased competition at the perceptual level and prioritized processing for arousing stimuli, this can scarcely explain the results of the LTM task. This is because only pairs that were correctly processed in the WM task were considered in the LTM task analyses, thereby equating WM performance. Hence, the “detrimental” effect of arousal on the LTM task can hardly be attributed to processes occurring during initial perception and encoding, but may be due to post-encoding/consolidation processes. However, one may argue that (some) pairs consisting of a high-arousal picture may have been less well processed during the WM task, without actually affecting performance on the WM decision task. Hence, whereas these relatively lower-level representations might have been sufficient to make an accurate WM decision, performance on the LTM task may have suffered [59]. On the other hand, other studies also suggest that processes after initial perception and encoding play a role in compromised LTM performance. For instance, a number of studies demonstrated impaired LTM for neutral items that occurred in temporal or spatial proximity to an arousing item, even when the temporal distance is as long as 6 seconds [16]. Moreover, Pierce & Kensinger (2011; [11]) reported two separate experiments that suggested differential consolidation processes for memory of word pairs consisting of emotionally positive relative to negative words. Furthermore, the fact that we found differential results for our WM and LTM binding task (with a significant Valence × Arousal interaction for the former and only a main effect of Arousal for the latter) provides additional evidence that post-encoding processes affect LTM performance. Finally, these results are in line with the proposal that negative affective valence may impair associative binding after a short delay, but improve binding processes after a longer delay [11]. This issue can be explored further by increasing the interval between WM and LTM task, where one could expect that memory for picture pairs consisting of negative pictures may be superior to memory for positive pictures as the interval increases.

On a final note, it may seem remarkable that performance on the binding LTM task was overall highly accurate; with an average proportion correct of 87.3%. This may be due to three reasons: Firstly, performance on two-alternative forced choice tasks is known to be more reliable and accurate than on yes/no recognition memory tasks [60]. Secondly, next to retrieving the relevant memory episode, participants might have remembered their own prior performance during the WM task which could serve as an additional cue, possibly improving memory performance. Finally, matching pairs of the LTM binding task were presented twice before, namely during the encoding and probe phase of the WM task whereas non-matching pairs of the LTM task were, as a pairing as such, not shown before. These double encodings are a consequence of administering both a WM and a LTM task (see [61] for a more detailed discussion. It is rather complex to circumvent this problem in a two-alternative-forced-choice associative recognition memory task and with the current setup of combining a WM and a LTM task, since both tasks would need to be changed. One option would be to change the LTM task in a way that the cue stimulus is presented together with the target stimulus, previously being paired with the cue during the *encoding phase* of the WM task, and the foil stimulus, previously being paired with the cue during the *probe phase* of the WM task. However, this would not only make the task a rather complex task in which participants would be required to reject the most recently experienced stimulus pairs and, thus, make the task essentially a source memory task. Also, applying a “correction” for WM performance, as employed in the present paper, would be nearly impossible since the ‘cue-target’ pairing would not have been probed during the WM task. A second alternative would be to leave some pairs unprobed during the WM task but instead test them later in the LTM task. This approach, however, would also render the applied “WM performance correction” complex. In addition, it would require a larger number of trials and/or more stimuli, which was not feasible with the present setup of the experiment and the IAPS stimulus database. Nevertheless, future studies could evaluate the effect of these double encodings by comparing the three alternatives in an appropriate study design.

### Conclusion

In summary, we demonstrated negative effects of arousal on both an inter-item WM and inter-item LTM task, using identical stimuli and highly similar task demands for these two kinds of tasks. Whereas attentional processes may explain the “detrimental effects” of arousal on WM performance, lower performance on the LTM task is likely due to post-encoding processes, differentially affecting pairs consisting of positive and negative stimuli. Thus, at least some effects may not be generalized across valence levels, supporting the view that valence, the type of task and the interval between study and test needs to be considered when studying the effects of “emotion” on associative memory.
